# Diagnostic Accuracy of Sputum Microscopy in Comparison With GeneXpert in Pulmonary Tuberculosis

**DOI:** 10.7759/cureus.11383

**Published:** 2020-11-08

**Authors:** Masab Umair, Shajee Ahmad Siddiqui, Muhammad Aadil Farooq

**Affiliations:** 1 General Medicine, Shaheed Zulfiqar Ali Bhutto Medical University (SZABMU) / Pakistan Institute of Medical Sciences (PIMS), Islamabad, PAK; 2 Specialty Medicine, Worcestershire Royal Hospital, Worcester, GBR

**Keywords:** tb, xpert mtb/rif, genexpert, acid fast bacilli, afb, zn smear microscopy, bcg, tuberculosis

## Abstract

Background

Tuberculosis (TB) is a major health problem. In Pakistan, the diagnosis of pulmonary tuberculosis mainly relies on acid-fast bacilli (AFB) smear microscopy and Xpert® MTB/RIF assay (Cepheid Inc., Sunnyvale, CA) - a nucleic acid amplification test - where available. There is a wide variation in the reported sensitivity of Ziehl-Neelsen (ZN) smear microscopy across previous studies. This study aimed to determine the accuracy of sputum ZN smear microscopy in diagnosing pulmonary tuberculosis as compared with the sputum GeneXpert (Xpert MTB/RIF assay) as the reference test.

Materials and methods

This is a retrospective cross-sectional study conducted in the outpatient department of the Pakistan Institute of Medical Sciences. This study included 326 patients, aged 12 years and above, who had their sputum samples tested for ZN smear microscopy and GeneXpert during the period January to June 2019. Patients' demographic details, sputum ZN smear microscopy, and GeneXpert test results were collected for data analysis. A case of pulmonary tuberculosis was defined as a patient with positive sputum GeneXpert test result.

Results

Out of the 326 patients, GeneXpert detected MTB deoxyribonucleic acid (DNA) in 50 patients and ZN smear microscopy detected AFB in 30 patients. There was a marginal male predominance among GeneXpert positive cases. Adolescents were the least affected age group. The sensitivity, specificity, positive predictive value (PPV), negative predictive value (NPV), and accuracy of ZN smear microscopy were 60%, 100%, 100%, 93.24%, and 93.87%, respectively. The positive likelihood ratio was infinite whereas the negative likelihood ratio was 0.4. The area under curve (AUC) for ZN smear microscopy was 0.800 and receiver operating characteristic (ROC) curve analysis revealed a diagonal straight line closer to the left upper corner.

Conclusion

Sputum ZN smear microscopy is a highly specific but moderately sensitive test for the diagnosis of pulmonary tuberculosis. This study recommends the sputum GeneXpert MTB/RIF test to avoid a missed diagnosis of smear-negative pulmonary TB.

## Introduction

Tuberculosis (TB) is an infectious disease caused by Mycobacterium tuberculosis (MTB). The lung is the most common site of the infection [1]. Transmission is via aerosol generated during cough [1]. TB is a preventable and treatable disease. Untreated TB causes significant morbidity and mortality. TB is a major health problem. According to the World Health Organization (WHO) Global TB report 2019, there were 10,000,000 new cases of TB globally, with 1,240,000 human immunodeficiency virus (HIV)-negative TB deaths in the year 2018 [2]. Pakistan is among the 30 highest TB burden countries. There were 562,000 new cases of TB, 28,000 drug-resistant TB cases and 43,000 HIV-negative TB deaths in the year 2018 in Pakistan [2].

Mycobacterial culture is the gold standard test for the diagnosis of TB, and it can detect as low as 10 bacilli/ml of the specimen [3]. However, mycobacterial culture is time-consuming and requires specialized laboratory infrastructure [3]. A GeneXpert Mycobacterium tuberculosis/rifampicin (MTB/RIF) assay (a nucleic acid amplification test) is a sensitive but expensive test for the diagnosis of TB with a lower detection threshold of 136 bacilli/ml of the specimen [3]. Additionally, a GeneXpert MTB/RIF assay can simultaneously detect the presence of rifampicin resistance, which is a surrogate marker of multidrug-resistant (MDR) TB [3]. However, the GeneXpert MTB/RIF assay costs 10 times more than the Lowenstein-Jensen (LJ) culture and 20 times as compared to ZN smear microscopy [4]. Acid-fast bacilli (AFB) smear microscopy is an inexpensive but less sensitive test for the diagnosis of TB with a lower detection threshold of 5,000 bacilli/ml of the specimen [3]. Ziehl-Neelsen (ZN; conventional light microscopy) and fluorochrome (light-emitting diode (LED) fluorescent microscopy) are the techniques used for AFB smear microscopy [3]. Fluorescent microscopy is a more rapid but more expensive technique as compared to Ziehl-Neelsen (ZN) smear staining, which is less expensive and widely available [5].

National Guidelines for the Control of Tuberculosis in Pakistan recommend the use of AFB smear microscopy for the diagnosis of pulmonary TB in adult non-immunocompromised patients not at risk of drug-resistant TB and in all patients with a presumptive diagnosis of TB, where GeneXpert MTB/RIF testing is not available [3]. GeneXpert MTB/RIF assay is recommended after microscopy in AFB smear-positive cases and in AFB smear-negative cases, with chest X-ray changes suggestive of TB [3]. The GeneXpert MTB/RIF assay is also recommended as an initial test for the diagnosis of presumed TB in children, immunocompromised patients, suspected extrapulmonary TB, suspected drug-resistant TB, and in patients with suggestive chest X-ray changes [3]. Mycobacterial culture is not recommended as an initial diagnostic investigation. However, mycobacterial culture is essential for the management of drug-resistant tuberculosis [3]. There are 22 culture laboratories in Pakistan set up by the National TB Control Programme [3].

However, there is wide variation in the sensitivity of ZN smear microscopy, ranging from 20% to 80% across various studies [5-7], whereas, the diagnostic performance of the GeneXpert MTB/RIF assay is comparable to that of mycobacterial culture [3]. In addition, the GeneXpert MTB/RIF assay outperformed mycobacterial culture for the detection of MTB in sputum samples in a prospective study by Shi et al. [8]. WHO recommends sputum GeneXpert MTB/RIF testing for all suspected cases of pulmonary tuberculosis [9]. However, because of resource limitations, the diagnosis of pulmonary TB in Pakistan relies mainly on AFB smear microscopy and GeneXpert MTB/RIF testing where available [3].

This study aimed to determine the accuracy of sputum ZN smear microscopy in diagnosing pulmonary tuberculosis as compared with the sputum GeneXpert MTB/RIF assay as the reference test.

## Materials and methods

This is a retrospective cross-sectional study conducted in the outpatient department of the Pakistan Institute of Medical Sciences. This study included 326 patients, aged 12 years and above, who had their sputum samples tested for ZN smear microscopy and GeneXpert MTB/RIF during the period from January to June 2019. Nonprobability consecutive sampling was used. Patients' demographic details, sputum ZN smear microscopy, and GeneXpert MTB/RIF test results were collected for data analysis. A case of pulmonary TB was defined as a patient with a positive sputum GeneXpert MTB/RIF test result. The diagnostic test performance of ZN smear microscopy was evaluated taking GeneXpert MTB/RIF as the reference investigation. A 2 x 2 contingency table was used for comparison. Frequencies were determined for true positives (TP), true negatives (TN), false positives (FP), and false negatives (FN). Sensitivity, specificity, positive predictive value (PPV), negative predictive value (NPV), positive likelihood ratio (LR+), negative likelihood ratio (LR-), and accuracy were calculated using the standard formulae. Receiver operating characteristic (ROC) curve analysis was performed and the area under curve (AUC) was determined.

Sensitivity: True Positive / (True Positive + False Negative) Specificity: True Negative / (True Negative + False Positive) Positive-Predictive Value (PPV): True Positive / (True Positive + False Positive) Negative-Predictive Value (NPV): True Negative / (False Negative + True Negative) Accuracy: True Positive + True Negative / (True Positive + False Negative + False Positive + True Negative) Positive Likelihood Ratio (LR+): Sensitivity / (1 - Specificity) Negative Likelihood Ratio (LR-): (1 - Sensitivity) / Specificity

## Results

A total of 326 patients were included in the study. Among these patients, 190 were males and 136 were females (Table 1). The mean age was 45 years (range: 13-92 years). Sputum GeneXpert MTB/RIF detected MTB DNA in 50 (15.3%) samples, whereas sputum ZN smear microscopy detected AFB in 30 (9.2%) samples. Among 50 GeneXpert MTB/RIF positive cases, 26 (52%) were males and 24 (48%) were females (Table 1). Adolescents (12-17 years) were the least affected age group, with only one GeneXpert MTB/RIF positive case (Table 1). 

**Table 1:** Gender and age group distribution of GeneXpert positive cases

	GeneXpert Positive (n)	Samples tested (n)
Distribution by Gender		
Males	26	190
Females	24	136
	50	326
Distribution by Age Group		
Adolescents (12-17 years)	1	15
Young adults (18-35 years)	18	108
Middle-aged adults (36-55 years)	14	93
Older adults (55 years and above)	17	110
	50	326

ZN smear microscopy and GeneXpert MTB/RIF test results are summarized in Table 2.

**Table 2:** Summary of ZN smear microscopy and GeneXpert test results ZN: Ziehl-Neelsen

Summary of the test results			
ZN smear microscopy			
Positive	30	Quantification	
		Scanty	9
		1+	10
		2+	7
		3+	4
Negative	296		
GeneXpert			
Positive	50	Quantification	
		Very Low	9
		Low	13
		Medium	11
		High	10
		Not Available	7
Negative	276		

**Figure 1:**
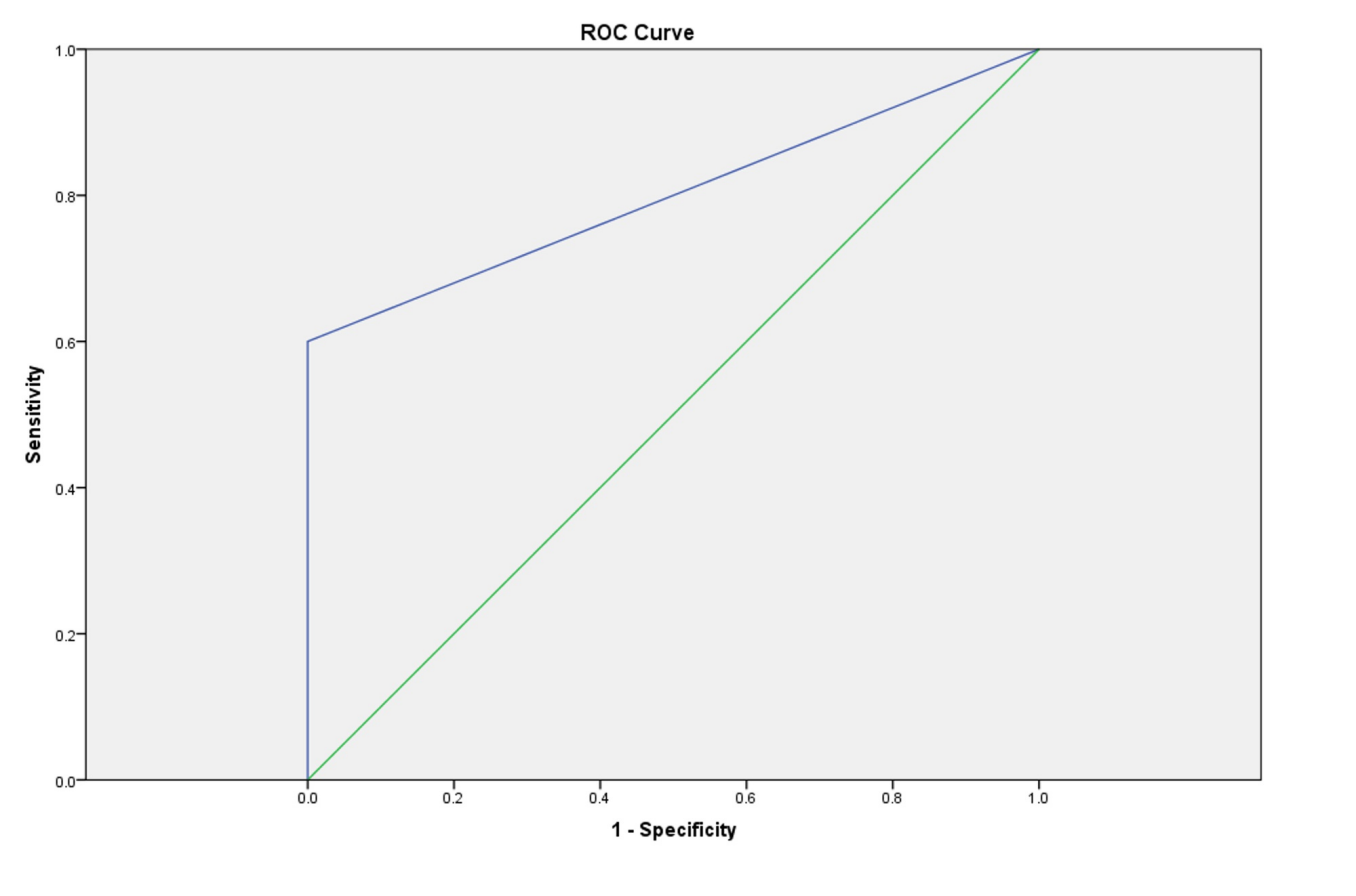
ROC curve for ZN smear microscopy taking GeneXpert as the reference test ROC: receiver operating characteristic; ZN: Ziehl-Neelsen

Diagnostic test performance, measures of diagnostic accuracy, and receiver operating characteristic (ROC) curve for ZN smear microscopy in comparison with GeneXpert MTB/RIF as the reference test are summarized in Table 3 and Figure 1.

**Table 3:** Diagnostic test performance of ZN smear microscopy taking GeneXpert as the reference test ZN: Ziehl-Neelsen

Diagnostic test performance of ZN smear microscopy		
2 × 2 contingency table		
	GeneXpert	
	Positive	Negative
ZN Smear Microscopy		
Positive	TP: 30	FP: 0
Negative	FN: 20	TN: 276
Measures of Diagnostic Accuracy of ZN Smear Microscopy		
Sensitivity (%)	60	
Specificity (%)	100	
Positive-Predictive Value (%)	100	
Negative-Predictive Value (%)	93.24	
Accuracy (%)	93.87	
Positive Likelihood Ratio	Infinite	
Negative Likelihood Ratio	0.4	
Area Under Curve	0.800	

## Discussion

Tuberculosis (TB) is a major health problem [2]. Although mycobacterial culture is the gold standard test for the diagnosis of TB, the diagnosis of TB in Pakistan relies mainly on AFB smear microscopy and GeneXpert MTB/RIF assay where available [3]. However, there is a wide variation in the reported sensitivity of ZN smear microscopy, ranging from 20% to 80% [5]. On the other hand, the sensitivity of the GeneXpert MTB/RIF assay is comparable to that of mycobacterial culture [3,8]. ZN smear microscopy and GeneXpert MTB/RIF are available at the Pakistan Institute of Medical Sciences (PIMS) where this study was conducted. This study has evaluated the diagnostic accuracy of sputum ZN smear microscopy in comparison with sputum GeneXpert MTB/RIF as the reference test.

In this study, males marginally outnumbered females among GeneXpert MTB/RIF positive cases. The WHO Global TB report 2019 and Qadeer et al. also reported a higher male preponderance among TB cases in Pakistan [2,10]. The analysis of age group distribution of GeneXpert MTB/RIF positive cases revealed the lowest prevalence in adolescents and the highest prevalence in older adults. Qadeer et al. reported a higher TB prevalence in the elderly and suggested the reactivation of TB in the presence of a weaker immune system as a possible explanation for this age trend [10]. There are studies supporting the efficacy of neonatal Bacillus Calmette-Guérin (BCG) vaccination against TB for 15 to 20 years [11]. BCG vaccination coverage is 80% in Pakistan [12]. We suggest the protective effects of BCG vaccination as a possible explanation for the lower TB prevalence in adolescents in our study. We recommend further studies to evaluate the protective effects of childhood BCG vaccination against pulmonary TB.

In our study, sputum ZN smear microscopy had moderate sensitivity but high specificity, positive-predictive value, and negative-predictive value taking the sputum GeneXpert MTB/RIF assay as the reference test. Mavenyengwa et al. reported slightly lower sensitivity but comparable specificity, positive-predictive value, and negative-predictive value of ZN smear microscopy [13]. The positive likelihood ratio for ZN smear microscopy was infinite in our study, suggesting the inevitable presence of pulmonary TB if the test result is positive. However, the negative likelihood ratio was 0.4; negative ZN smear microscopy reduces but does not completely eliminate the probability of the presence of pulmonary tuberculosis. ROC curve analysis of sputum ZN smear microscopy revealed a straight diagonal line closer to the left upper corner and an area under the ROC curve of 0.800, suggesting its ability to discriminate diseased (sputum GeneXpert positive pulmonary TB cases) from non-diseased (sputum GeneXpert negative cases). Mavenyengwa et al. reported a similar ROC curve but with marginally lower AUC [13].

Sputum ZN smear microscopy missed 20 (16.4%) cases when compared with the sputum GeneXpert MTB/RIF test in our study. Other authors also had similar findings [1,5,13-16]. An explanation for the lower sensitivity of AFB smear microscopy is the higher detection threshold in comparison with the GeneXpert MTB/RIF assay, i.e. 5,000 bacilli/ml vs 136 bacilli/ml of the specimen, respectively [3]. A missed diagnosis of pulmonary tuberculosis prolongs morbidity in the affected individual. Although smear-negative cases of pulmonary TB are considered less infectious than smear-positive cases, smear-negative cases can still transmit the disease. It has been estimated that 10%-20% of TB transmission is from smear-negative cases of pulmonary TB [17]. Furthermore, it has been estimated that the average patient cost of tuberculosis is $847, including medical expenses (20%), non-medical expenses (20%), and loss of income (60%) in low and middle-income countries [18]. On average, this cost approximates 58% of the annual individual income [18]. Therefore, a missed diagnosis of smear-negative pulmonary TB can have significant financial implications for the individuals, families, and the country as a whole. The GeneXpert MTB/RIF assay is a sensitive and specific test for the diagnosis of smear-negative culture-positive pulmonary tuberculosis [19]. Although the GeneXpert MTB/RIF assay is more expensive than ZN smear microscopy, we still recommend GeneXpert testing where available to avoid prolonged morbidity in the affected individuals and to avoid the direct and indirect costs of missed diagnosis of smear-negative pulmonary TB.

## Conclusions

Sputum ZN smear microscopy is a highly specific but moderately sensitive test for the diagnosis of pulmonary tuberculosis. This study recommends the sputum GeneXpert MTB/RIF test in preference to sputum ZN smear microscopy for the diagnosis of pulmonary tuberculosis to avoid a missed diagnosis of smear-negative pulmonary tuberculosis. We also recommend further studies to evaluate the protective effects of childhood BCG vaccination against tuberculosis.
